# HIV-1 is Transported into the Central Nervous System by Trafficking Infected Cells

**DOI:** 10.20411/pai.v7i2.524

**Published:** 2023-01-23

**Authors:** Laura P. Kincer, Gretja Schnell, Ronald Swanstrom, Melissa B. Miller, Serena Spudich, Joseph J. Eron, Richard W. Price, Sarah B. Joseph

**Affiliations:** 1 Lineberger Comprehensive Cancer Center, University of North Carolina at Chapel Hill, Chapel Hill, NC; 2 UNC Center for AIDS Research, University of North Carolina at Chapel Hill, Chapel Hill, NC; 3 Department of Microbiology and Immunology, University of North Carolina at Chapel Hill, Chapel Hill, NC; 4 Department of Biochemistry and Biophysics, University of North Carolina at Chapel Hill, Chapel Hill, NC; 5 Department of Pathology & Laboratory Medicine, University of North Carolina at Chapel Hill, Chapel Hill, NC; 6 Division of Neurological Infections and Global Neurology, Department of Neurology, Yale University, New Haven, CT; 7 Division of Infectious Diseases, Department of Medicine, University of North Carolina at Chapel Hill, Chapel Hill, NC; 8 Department of Neurology, University of California at San Francisco, San Francisco, CA

**Keywords:** HIV, HCV, NeuroAIDS, CNS, blood-brain barrier, blood-cerebrospinal fluid barrier, viral trafficking

## Abstract

**Background:**

In this work, we carried out a cross-sectional study examining HIV-1 and HCV free virus concentrations in blood and cerebrospinal fluid (CSF) to determine whether HIV-1 enters the central nervous system (CNS) passively as virus particles or in the context of migrating infected cells. If virions migrate freely across the blood-cerebrospinal fluid barrier (BCSFB) or the blood-brain barrier (BBB) then HCV and HIV-1 would be detectable in the CSF at proportions similar to that in the blood. Alternatively, virus entry as an infected cell would favor selective entry of HIV-1.

**Methods:**

We measured HIV-1 and HCV viral loads in the CSF and blood plasma of 4 co-infected participants who were not on antiviral regimens for either infection. We also generated HIV-1 *env* sequences and performed phylogenetic analyses to determine whether HIV-1 populations in the CSF of these participants were being maintained by local replication.

**Results:**

While CSF samples taken from all participants had detectable levels of HIV-1, HCV was not detectable in any of the CSF samples despite participants having HCV concentrations in their blood plasma, which exceeded that of HIV-1. Further, there was no evidence of compartmentalized HIV-1 replication in the CNS (Supplementary Figure 1). These results are consistent with a model where HIV-1 particles cross the BBB or the BCSFB within infected cells. In this scenario, we would expect HIV-1 to reach the CSF more readily because the blood contains a much greater number of HIV-infected cells than HCV-infected cells.

**Conclusions:**

HCV entry into the CSF is restricted, indicating that virions do not freely migrate across these barriers and supporting the concept that HIV-1 is transported across the BCSFB and/or BBB by the migration of HIV-infected cells as part of an inflammatory response or normal surveillance.

## INTRODUCTION

The blood-cerebrospinal fluid barrier (BCSFB) and blood-brain barrier (BBB) maintain proper brain function and central nervous system (CNS) homeostasis by restricting the entry of pathogens, proteins, and cells into the CNS [1, 2]. Yet, HIV-1 can be detected in the cerebrospinal fluid (CSF) within a few weeks of transmission [3] and, while HIV-1 concentrations are typically lower in the CSF than in the blood plasma, HIV-1 is detectable in the CSF of the vast majority of untreated people living with HIV (PLWH) [4].

The presence of HIV-1 in the CSF is sometimes due to viral replication within the CNS [5]. There are 3 general sites in the CNS where HIV-1 may replicate: immune cells within the CSF, tissue proximal to the CSF (eg, meninges or choroid plexus), or the brain parenchyma. Replication at the first 2 sites may allow virions to efficiently seed the CSF. In contrast, in order to reach the CSF, viruses replicating in the brain parenchyma may require transport by interstitial fluid (ISF), of which approximately 15% drains into the CSF [6]. Previous studies have observed that the CSF can contain lineages of HIV-1 [7–9] or SIV [10] that are not present in the blood, a pattern generated by sustained viral replication locally within the CNS. However, the specific sites of viral replication in the CNS remain poorly understood.

While sustained viral replication in the CNS produces lineages that are genetically distinct from populations in the blood (ie, compartmentalized), the alternative state is when genetic analysis of CSF virus suggests that CSF virus is not produced by local replication. In these cases, HIV-1 detected in the CSF is genetically well mixed with viral populations in the blood, indicating that the viral population in the CSF is produced by migration from the blood into the CSF with little to no local replication. Migration into the CSF could occur at the choroid plexus with direct entry into the CSF (ie, crossing the BCSFB) [1]. Alternatively, it could occur at cerebral blood vessels in the brain parenchyma (ie, crossing the BBB) followed by movement via the ISF to the CSF. Addressing how virus crosses one or both of these barriers and sustains a detectable CSF viral population in the absence of local replication is complicated by the fact that virus in the blood can exist as free virus particles, in infected circulating immune cells (primarily CD4+ T cells), or occasionally as virions bound to dendritic cells (DCs).

In this study we explore whether HIV-1 in the blood enters into the CSF passively as a virus particle or in infected cells trafficking into the CNS as part of inflammation or normal surveillance. While both hepatitis C virus (HCV) and HIV-1 viral participles can reach high levels in the blood [11], the viruses differ in their target cells with HCV replicating in hepatocytes in the liver and HIV-1 primarily replicating in CD4+ T cells in lymphoid tissue and in the blood. Further, given that HCV particles are half the size of HIV-1 particles (~60 nm vs 120 nm [12]), HCV should passively enter the CNS as efficiently or more efficiently than HIV-1 if entry is by a passive diffusion mechanism of virus particles. To test the 2 most likely models of virus movement from the blood/lymphoid tissue into the CNS, we leveraged the fact that HIV-1 and HCV virions are both present in the blood of co-infected people, have similar properties of being enveloped viruses, but that only HIV-1 commonly infects cells in the blood and lymphoid system ([13], but also see [14, 15]).

Model 1 proposes that free virus particles cross the BCSFB/BBB into the CSF. Model 2 proposes that virus is trafficked into the CSF via infected cells, a process whose rates will be governed by the frequency of infected cells in the blood. To test these models, we compared the viral loads (HIV-1 and HCV) and viral genetic diversity (HIV-1 only) in the blood and CSF, and barrier integrity in a small cohort of people co-infected with HIV-1 and HCV and not on antiretroviral therapy.

## METHODS

### Participants and Samples

Four HIV-1 and HCV co-infected participants were identified from 2 studies that included collection of blood plasma and CSF. We included 3 participants from the THINC study (PIDs 1019, 1023, and 100), an ongoing study to examine blood/CSF compartmentalization in people with CD4+ T-cell counts less than 400/uL in the blood (Joseph et al., in preparation). One additional participant (PID 7146) was included from a previously published primary HIV-1 infection study [16, 17]. All samples were collected with informed consent and under local IRB-approved protocols and as part of NIH-funded studies.

### Viral Load Determinations

Viral loads for HIV-1 and HCV in the blood and CSF were determined using Abbott RealTime assays for HIV-1 (lower limit of detection [18] of 40 RNA cp/mL) and HCV (LOD of 12 RNA IU/mL). According to World Health Organization guidelines [19], a conversion factor was used to convert HCV RNA copies/mL to international units (IU)/mL.

### Sequence Analysis

For 3 participants (1019, 1023, and 100), the MiSeq next generation sequencing (NGS) platform was used to determine the genetic relationship between HIV-1 RNA isolated from the blood and CSF. The NGS analysis of the V1-V3 region of the HIV-1 *env* gene was performed using our previously described Primer ID method [20]. To assess sequence compartmentalization versus sequence equilibration between the blood and CSF compartments, 50 sequences from each compartment were used to create a neighbor-joining tree. In addition, single genome amplification (SGA) and sequencing was previously used to generate HIV-1 blood and CSF sequences for participant 7146 [16].

## RESULTS

**Figure 1.**
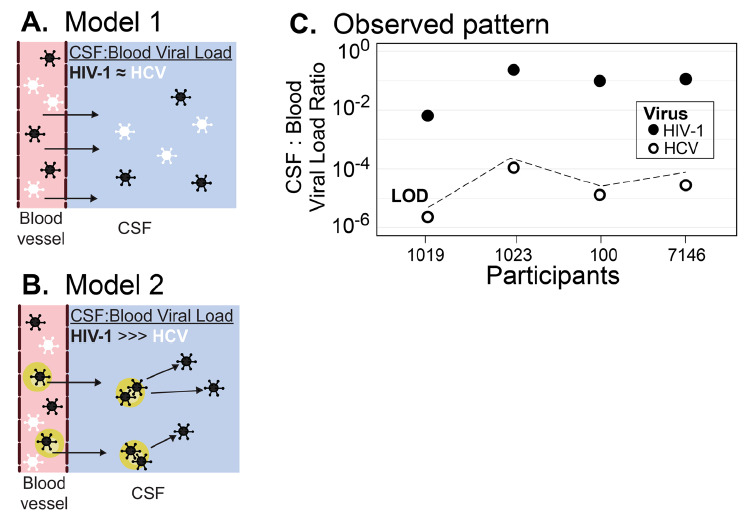
**HIV-1 is actively trafficked into the CNS by infected cells.** (A) Model of passive diffusion of virus into the CNS. (B) Model of virus trafficking into the CNS in infected cells. (C) HIV-1 has proportionally higher levels of virus in the CSF compared to HCV. The viral loads of HIV-1 and HCV in the blood and CSF are plotted as a ratio of CSF:Blood viral load for each of the 4 participants. Values for HIV-1 are shown in solid circles, for HCV in open circles. Because the HCV viral load was undetectable in the CSF, a value just below the ratio that would have been recorded at the limit of detection (LOD) is shown.

We sought to test 2 models of HIV-1 entry into the CNS, as reflected by virus in the CSF (Figure 1A and 1B). In order to do this, we used CSF and blood plasma samples collected from 4 participants who were co-infected with HIV-1 and HCV and not receiving therapy for either virus (Table 1). These participants had moderate CD4+ T-cell counts in the blood. Two of the participants had only low levels of white blood cells (WBC) in their CSF while the other 2 (PIDs 100 and 7146) had significant numbers of WBC in the CSF.

**Table 1.** 

Participant	Age	CD4 T cell count (cells/μL)	HIV-1 RNA			HCV RNA^b^			CSF WBC (cells/μL)	CSF RBC (cells/μL	CSF protein (mg/dL)	CSF: Serum albumin ratio^e^
			Blood VL log_10_	CSF VL log_10_	CSF: Blood ratio^a^	Blood VL log_10_	CSF VL log_10_	CSF: Blood ratio^d^				
1019	45	281	5.2	3.0	6×10^−3^	6.6	ND^c^	< 3×10^−6^	4	1	42	–
1023	29	394	3.9	3.3	3×10^−1^	5.0	ND^c^	< 1×10^−4^	5	1	22	–
100	30	324	5.8	4.7	8×10^−2^	5.9	ND^c^	< 2×10^−5^	23	4	57	7
7146	36	476	5.3	4.4	1×10^−1^	5.6	ND^c^	< 3×10^−5^	97	?	73	7

a The HIV-1 CSF viral load divided by the HIV-1 blood viral load

b HCV VL is reported in international units (IU) [19]

c ND - not detected with a limit of detection of 12 IU/mL

d The HCV ratio was calculated using the HCV LOD (12 IU/mL) as the maximum amount of HCV in the CSF

e The ratio of CSF to serum albumin x 10^3^

In order to determine whether these high CSF WBC counts were associated with BCSFB/BBB dysfunction, we measured the ratio of albumin in the CSF and blood and observed that both participants with available data had elevated levels (Table 1; see [21] for standard values). In addition, we found moderately (PID 100) to substantially (PID 7146) elevated CSF total protein levels in the same 2 participants (see [22] for standard values). Thus, there is evidence that these 2 participants had BBB/BCSFB dysfunction in conjunction with elevated CSF WBC counts.

HIV-1 RNA levels in the blood ranged from log_10_ 3.9 to 5.8 RNA cp/mL (Table 1). On average, the levels of HCV in the blood were approximately 10-fold higher (range log_10_ 5.0 to 6.6 RNA IU/mL). However, it is worth noting that aliquots of a control sample (ie, the international standard) with an agreed upon copy number were used to convert measures of HCV RNA/mL to RNA IU/mL [19]. No such standardization is used for estimates of HIV-1 RNA, making it difficult to precisely compare HCV RNA IU/mL to HIV-1 RNA/mL.

Despite some imprecision in this comparison, it is clear that concentrations of HCV in the blood were higher than that of HIV. The range of HIV-1 viral RNA levels in the CSF was 3.0 to 4.7 log_10_ RNA cp/mL and were consistently lower than the paired values in the blood, ranging between 0.6 to 2.2 log_10_ lower. The 2 participants with the highest HIV-1 RNA loads in the CSF (PIDs 100 and 7146) were also the 2 with evidence of BBB/BCFB dysfunction (ie, elevated CSF albumin) and elevated CSF WBC counts. Despite all participants having at least 3.0 log_10_ HIV-1 RNA cp/mL CSF and 2 participants having evidence of BBB/BCFB dysfunction, HCV RNA was undetectable in the CSF of all 4 participants.

**Figure 2.**
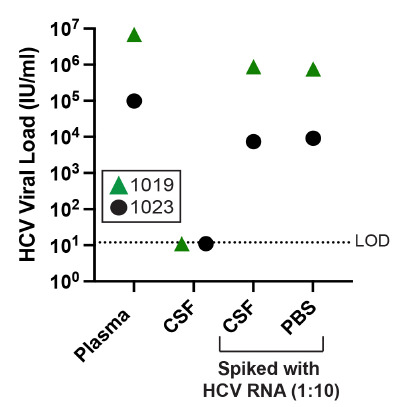
**HCV is detectable in spiked CSF and PBS.** HCV+ blood from 2 participants, 1019 (green triangles) and 1023 (black circles), was used to spike patient matched CSF as well as PBS. Ten percent plasma was mixed with 90% CSF or 90% PBS, and an HCV viral load was performed on these spiked samples along with pure plasma and pure CSF. The limit of detection (LOD) for the HCV VL assay is shown with a dashed line.

Since HCV was undetectable in all CSF samples, a CSF to blood viral load ratio for HCV was calculated as the limit of detection of the HCV viral load assay (12 RNA IU/mL) divided by the observed HCV serum viral load. As can be seen in Figure 1C, HCV CSF:blood viral load ratios were more than 10^3^-fold lower than the ratio for HIV-1. Moreover, HCV was detectable in CSF and PBS when these fluids were spiked with HCV+ blood plasma (Figure 2) suggesting the negative HCV values observed in the CSF are real and not due to inhibitors in the fluid or limitations of the assay. This result indicates that HCV migration into the CNS is highly restricted, and we infer that HIV-1 achieves detectable concentrations in the CSF by a mechanism that is not available to HCV, namely the trafficking of infected cells.

Finally, we used compartmentalization analysis to confirm that the populations of HIV-1 in the CSF of these 4 participants were not being maintained by local CNS replication (a process that could have inflated the concentration of HIV-1 in the CSF). As can be seen in Supplementary Figure 1, the HIV-1 populations in the blood and CSF for these participants were well mixed (ie, largely equilibrated) and are thus primarily being maintained by migration into that compartment and not the result of local replication.

## DISCUSSION

The presence of elevated levels of HIV-1 in the CSF beyond what can be accounted for by a nonspecific mechanism that would also allow HCV to enter the CNS suggests that HIV-1 enters the CSF/CNS through the trafficking of infected cells, most likely CD4+ T cells (See [23]). For these participants, there was no evidence of local viral HIV-1 replication in the CNS at the timepoints surveyed, which would have appeared as a compartmentalized viral lineage; therefore, it is necessary to account for the presence of HIV-1 in the CSF by mechanisms that would exclude HCV. It is, however, worth noting that 1 participant (7146) did have compartmentalized lineages in their CSF at a later timepoint [16], but our results indicate that at the timepoint surveyed in this study, viral replication in the CNS was either occurring at levels below the limit of detection and/or was obscured by an elevated virus load caused by the influx of infected cells, associated with pleocytosis.

A previous study of individuals coinfected with HIV-1 and HBV [24] also found evidence that the BCSFB/BBB restricts passage of free virions into the CNS. In that study [24], individuals with detectable HIV-1 and HBV in their blood and no history of antiretroviral therapy, had CSF to blood viral load ratios that were on average 6000X lower for HBV than HIV-1. Both HCV and HBV are restricted to replication in hepatocytes [25] and are therefore unlikely to be trafficked to the CNS as infected cells. Further, given that HCV and HBV particles are approximately half the size of HIV-1 particles (~60 nm [12] and 45 nm [26], respectively, vs 120 nm, [12]), there is no reason to think that the BCSFB/BBB provides a size barrier that allows the passage of free HIV-1 virions, but not free HCV or HBV virions.

An additional process that could transport virus into the CSF is viral transport by dendritic cells (DCs). Both HIV-1 [27] and HCV [28] are known to bind C-type lectin receptors on the surface of DCs. Further, DCs have been observed to migrate into the CNS more often during some diseases [29–31], including SIV infection [32]. While we cannot rule out that in our study HIV-1 virions were trafficked across the BBB or BCSFB by DCs, this mechanism would have been available to both HIV-1 and HCV, yet we did not detect HCV in the CSF. Thus, trafficking of virus across the BBB/BCSFB by DCs did not appear to be a key mechanism in our participants.

A surprising observation in this study is that HCV was undetectable in the CSF of 2 participants with evidence of BBB/BCSFB dysfunction, suggesting that this dysfunction did not compromise these barriers sufficiently to allow HCV virions to freely cross at detectable levels. In contrast, studies examining neurosymptomatic cohorts with extremely low CD4+ T-cell counts and CNS coinfections (eg, toxoplasmosis, CMV encephalitis, cryptococcosis, etc.) have reported HCV in CSF [14, 15, 33, 34] and brain tissue [15, 34]. HCV has also been reported in the CSF of individuals with cognitive dysfunction but without severe liver disease or HIV-1 coinfection [34]. This evidence suggests that, in a subset of people, HCV may enter the CNS more readily. This is possibly due to profound disruption of the BBB or BCSFB by CNS coinfections or other complications associated with severe immunosuppression or cognitive impairment. However, it is clear that, in the participants in our study, HCV was not entering the CSF and therefore could serve as an internal control for passive diffusion of virus particles across the BBB.

Our inference that HIV-1 is trafficked into the CNS by infected cells does not distinguish which cell type is infected. Two observations support our interpretation that infected CD4+ T cells traffic into the CSF/brain and release HIV-1. First, as we state above, studies of HIV-1 cellular tropism suggest that CD4+ T cells are the most commonly infected cells in the blood (reviewed by [35]) and thus the most likely to carry HIV-1 into the BCSFB/BBB. Second, a previous cross-sectional study of neuroasymptomatic people observed that people with very low levels of CD4+ T cells in the blood (<50 cells/uL) have CSF viral loads that are approximately 1 log_10_ lower than that of people with higher CD4+ T-cell counts but comparable plasma viral loads [36]. The interpretation is that with fewer CD4+ T cells available to traffic to the CNS the CSF virus level declines. These observations indicate that infected CD4+ T cells that traffic from the blood into the CSF/CNS continuously expose this tissue to HIV-1. This is also consistent with previous cross-sectional studies showing a positive association between CSF WBC and CSF VL [4, 37–39], although replication in trafficking cells after they reach the CNS could also amplify virus in that compartment. Finally, we think it is unlikely that virus is being introduced into the CNS by the migration of infected monocytes. The virus found in the blood requires a high density of CD4 for efficient entry, which is found only on CD4+ T cells and not on monocytes [40]. This makes infected CD4+ T cells the likely candidate for transport of HIV-1 into the CSF in the absence of local replication within the CNS.

## SUPPLEMENTARY DATA

Supplementary materials are available at the *Pathogens and Immunity* website. Supplementary data may be provided by the authors to benefit the reader. Supplementary data are not copyedited and are the sole responsibility of the authors. Questions or comments related to supplementary materials should be addressed to the corresponding author.
