# Supplementary material for: HIV-1 is Transported into the Central Nervous System by Trafficking Infected Cells
Source: Pathog Immun. 2023 Jan 23;7(2):131–42. doi: 10.20411/pai.v7i2.524 (PMC9973728; doi:10.20411/pai.v7i2.524)
Supplement: Supplementary Figure 1 [file pai-7-131-s01.pdf]

PATHOGENS AND IMMUNITY
IN ASSOCIATION WITH CASE WESTERN RESERVE UNIVERSITY AND THE RICHARD J. FASENMYER FOUNDATION

SUPPLEMENTARY FIGURE 1

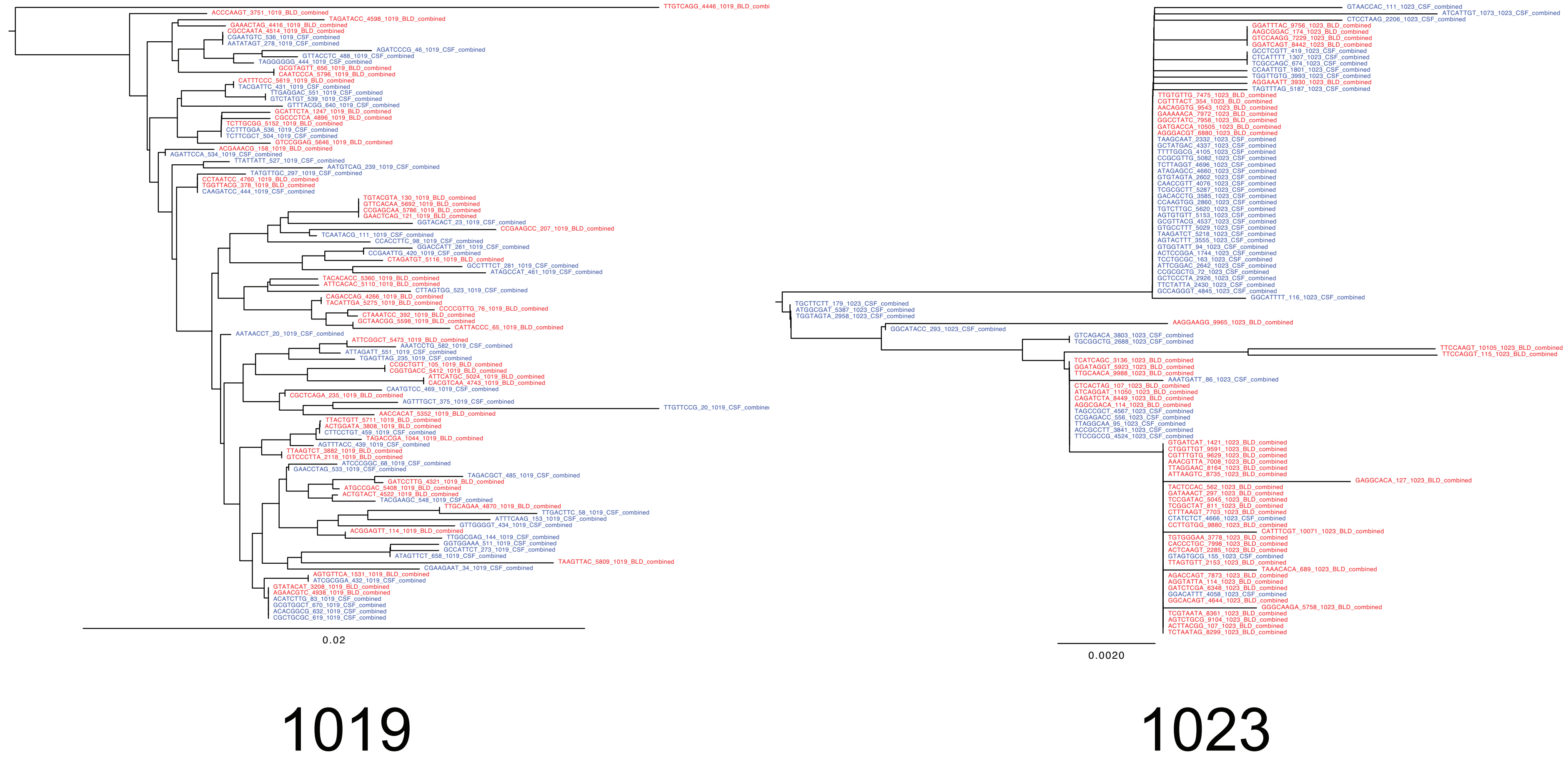

Kincer LP, Schnell G, Swanstrom R, Miller MB, Spudich S, Eron JJ, Price RW, Joseph SB. HIV-1 is Transported into the Central Nervous System by Trafficking Infected Cells. *Pathogens and Immunity*. 2023;7(2):131–142. doi: 10.20411/pai.v7i2.524

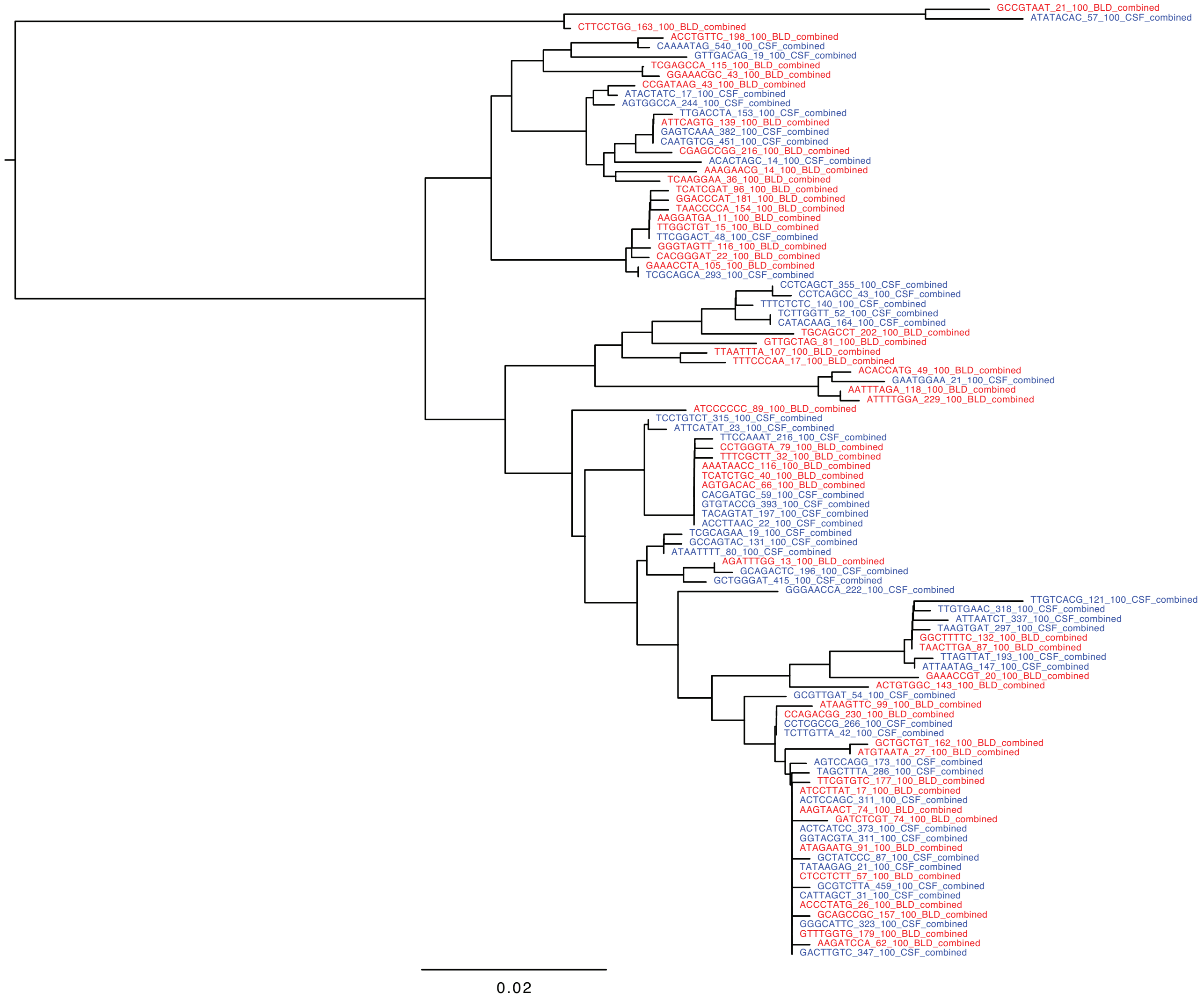

100

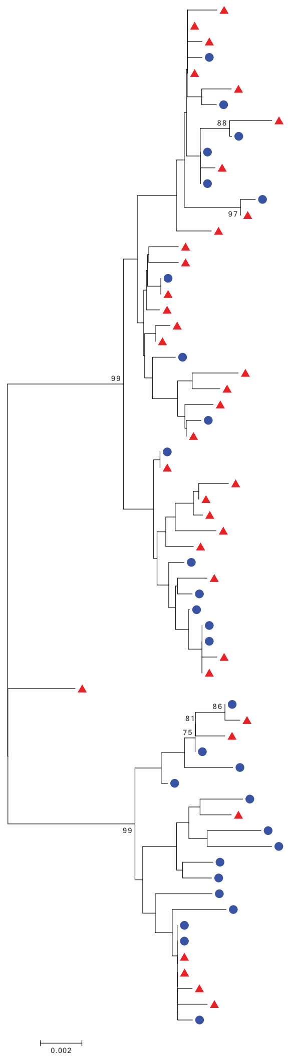

7146

Kincer LP, Schnell G, Swanstrom R, Miller MB, Spudich S, Eron JJ, Price RW, Joseph SB. HIV-1 is Transported into the Central Nervous System by Trafficking Infected Cells. *Pathogens and Immunity*. 2023;7(2):131–142. doi: 10.20411/pai.v7i2.524

PATHOGENS AND IMMUNITY
IN ASSOCIATION WITH CASE WESTERN RESERVE UNIVERSITY AND THE RICHARD J. FASENMYER FOUNDATION

Supplementary Figure 1. Sequence analysis of HIV-1 in blood and CSF. Viral RNA was extracted from plasma and CSF for each subject. For three participants (1019, 1023 and 100), RNA was copied into cDNA then amplified by PCR for a region spanning the V1 to V3 region of the *env* gene. Each RNA template was tagged with an individual Primer ID in the cDNA primer to allow the construction of a template consensus sequence for each starting RNA template. After amplification the PCR product was sequenced using the MiSeq platform. For one participant (7129), RNA was copied into cDNA and the full-length *env* gene was analyzed using our previously described SGA method [16]. All sequences were analyzed in a neighbor-joining tree where each branch is a single template consensus sequence. One tree is shown for each subject. Red: viral sequences from the blood plasma; blue: viral sequences from the CSF.
